# Network governance theory as basic pattern for promoting financial support system of the poor in Iranian health system

**DOI:** 10.1186/s12913-021-06581-3

**Published:** 2021-06-07

**Authors:** Manal Etemadi, Patrick Kenis, Kioomars Ashtarian, Hasan Abolghasem Gorji, Hannaneh Mohammadi Kangarani

**Affiliations:** 1 Department of Health Services Management, School of Health Management and Information Sciences, Iran University of Medical Sciences, Tehran, Iran; 2 Department of Public Governance, Tilburg University, Tilburg School of Economics and Management, Tilburg, Netherlands; 3 Department of Public Policy, School of Law and Political Sciences, University of Tehran, Tehran, Iran; 4 Department of Agriculture and Natural Resources, University of Hormozgan, Bandar Abbas, Iran

**Keywords:** Network governance, Financial protection, The poor, Health, Shared-governance

## Abstract

**Background:**

The share of out-of-pocket payments in Iranian families has the greatest burden on the poor and lead to an impoverishment caused by catastrophic health expenditures. In order to improve access of the poor to public resources, it is necessary to create a better governance system and effective policy-making. The purpose of this study is to improve network effectiveness of the Iranian health system and to design a financial protection network for the poor, based on the network governance theory.

**Methods:**

We are using a quantitative method framework in conjunction with a Social network analysis (SNA) strategy. To draw an optimal network, we conducted interviews with experts by focusing on the arrangement and relationship among different institutions. The research sample was purposefully selected. We used UCINET software for data analysis and NetDraw software to draw networks.

**Results:**

In this article, an optimal network was proposed with the following characteristics: First, the problem of the density of relationships among several central institutions and the isolation of the other institutions have been solved. Second, in our model, the relationships have been distributed in a balanced manner among all institutions in the network. Third, the number of participants has been reduced and consensus on poor people support policies has been achieved in this optimal network. Forth, executive organizations keep their central positions and upper institutions are not at the central position, so that the power is distributed in favor of more balanced governance. However, in order to increase efficiency and to have coherent decision-making, it is necessary to establish a “core” for this optimal network. The “core” has to include the organizations with the most relationship with others.

**Conclusion:**

The result revealed that the usefulness of network analysis as a tool for proposing the effectiveness of governance. By strengthening the relationship among the main actors, an organized system of network management can be achieved. The network has to include all actors from different levels, from policy-making to implementation. The network also has to clarify the tasks from identifying the poor to covering costs. From an academic perspective, this study showed the adequacy of network analysis as a tool for policy sciences. Governance in our optimal health financial protection model follows the shared-governance pattern due to its high density, low centralization and low distance. The model of network governance can be the source of changes in the health governance system. It is a necessary structural condition to provide access to universal health coverage.

## Introduction

Poor people bear a greater burden of health costs than the rich, and they don’t have adequate access to the health care services [1]. One in four low-income families is forced to burden heavy debts or sell its assets for treatment [2, 3]. In the absence of a targeted mechanism of government health subsidies for the poor, the rich are more beneficial than the riche. According to paragraph 10–4 of the policies enacted by the Expediency Council government has the responsibility “to accord special aid to the needy and the low-income deciles.

In the “Health Transformation Plan” this special aid contains user fee reduction for patients in public hospitals, which covers all citizens, not just the poor; as a result, the proportion of insured poor has decreased [4]. Iranian experience shows that insurance coverage without targeted mechanisms could lead to Adverse effects on redistribution of resources in the financing of health services and jeopardizes justice in financial access to health services [5]. Rationing, by restricting the needy groups’ visits only to the university affiliated centers, waiting queues and limiting services due to high referrals in government centers [6]. The most important step to provide financial access for poor is to implement reform in health care system with a clear approach to insist on institutional arrangement. Thus, institutional analysis can help for designing a policy network model for supporting the poor [7]. Good institutional network can identify effective institutions and key linkages for reducing poverty [8]. Policy design is challenging in Iran because of complexities of institutions, Overlapping the roles, diversity of responsibilities, and different methods of implementations. These issues need to be resolved in the way that the roles and responsibilities of key players be clarified and cooperative structures to be strengthened. In the same way cooperative procedures, management capacity building and consultation process need to be mapped [9].

Iran has 44 organizations/institutions that are required to comply with legal obligations. For supporting 27 categories of the poor to financially access health services. This plurality of institutions is unbalanced with regards to their linkages and has created challenges for coordination, responsibility, efficiency, and performance. For instance, for women-headed families and Orphans there is 19 organizations and for disabled persons 17 organizations are obliged to cooperate [10, 11].

In different examples, the governance deficit in Iran’s health system has been examined in communications between organizations involved in providing services. The presence of multiple institutions of stewardship and decision-making, as well as inefficiency in coordination and organization of various components within the health sector and between other sectors, pose a challenge to Iran’s health system stewardship. Bad communication and policy coordination between the health and social welfare sectors, as well as a lack of other institutions’ involvement and ineffective governance structures, have been implemented [12]. Financial protection necessitates intersectoral cooperation between governmental and non-governmental entities in charge, but cooperation among institutions is one of the policy challenges in Iran [13].

Iran’s fragmented health-care system has resulted in poor financial protection against medical expenses, high cost sharing, mission duplication, and a lack of accountability [14]. The same problem persists when it comes to other organizations’ coordination and collaboration in order to protect citizens financially. To efficiently serve the needy and achieve the mission of helping to improve their health, charitable organizations, for example, must coordinate and collaborate with counties, governorates, welfare organizations and relief councils, and medical universities. However, in the Iranian health system, such a partnership between these institutions has yet to be established [15].

Even though governance is a difficult issue in our health system, few studies have looked at the health financial protection system in Iran from an institutional and participation standpoint, despite the fact that we believe the majority of problems in health system support for the poor are rooted in the governance of Iran’s health system. As a result, this research attempted to take a fresh look at the issue.

### Network governance

Implementing public policies is a cooperative task and it is impossible to conceive the one organization without others. In a systematic perspective, organizations have different task and organized roles within a network and each of them has specific role [16]. complex policy problems need collective action and coordinative approach by different players [17].

The literature of public policy suggest that networks are a solution for complex problems. These problems are multicausal and have no clear definitions, our solutions for complex problems can cause side effects and can create conflicts among different governmental sectors [18].. Systemic solutions for complex problems need participation of different stakeholders; so that it necessitate multi-actor approach for policy problems. Recent literature shows a paradigm shift from hierarchical model of delivery service to networks [19].

Networks consist of different independent organizations that collaborate to achieve their social goals. These networks can be created by law or by a contract among them. They are considered as a formal collective action mechanism for accomplishment of the goals of several organizations -governmental as well as non-governmental or non for benefit organizations [20]. literature on policy analysis shows that in complex situations, puzzle solving as policy analysis method can discover solutions and designs. As Christopher Winship state policy analysis need a model of analysis to substitute instrumental rationality for resolving conflicting policy goals [21].

In the 1990s, a new method of network-based governance emerged that expanded the range of management and coordination mechanisms. This method has been accompanied by a critique of the previous two methods, namely market and hierarchy method, and is known for its wide chain of communication, informal organizational forms, and trust-based relationships [22].

The most popular approaches to health governance are creating intersectoral networks, hybrid organisations, and policy coordination, and network analysis is a tool for drawing communications in the organizational network. Individual organizations have been replaced as reform agents by networks, and networks are better suited to manage public benefits such as wellbeing [23].

For Provan and Kenis, network governance involves “the use of institutions and structures of authority and collaboration to allocate resources and to coordinate and control joint action across the network as a whole.” Therefore, network governance has two related dimensions of governance structure (collaboration structures) and governance mechanism (coordinative tasks in networks). There are three types of governance of networks structures: a shared-governance network (each network member is responsible for decision-making and management), a lead organization (a dominant organization responsible for network management), and a network administrative organization (creating an independent out of the network entity to manage it) [24].

After the classification of Provan and Kanis, the core-peripheral governance form was introduced, which is similar to the lead organization form, except that the responsibility of leading of the network lies not with one organization but with a number of network organizations. The logic for this kind of responsibility is that network consists of subgroups of organizations in different areas; it is not necessary for all of them to be involved in decision-making. The core consists representative of different organizations from each subgroup that can manage the network in the same way as a shared-governance form or a lead organization. This type of governance is the most effective form for complex issues that involve various sub-processes [25].

### Network analysis

The organizational network strategy is an excellent way to comprehend health-related problems. The information gleaned from network analysis is used to aid capacity building by forming a strong network of partner organizations. The health sector’s application of the organizational network methodology has reached maturity. If achieving a goal in the health sector exceeds an organization’s capability, network-level analysis is the most effective method for analyzing health issues and considers a focused and intelligent inter-sectoral mechanism for problem solving [26].

Four underlying factors act as key predictors of the effectiveness of network governance performance: the level of trust among members (density and centrality), the number of organizations in the network, the level of consensus on network goals and capacities of the network [27]. Network optimization as a policy design can be presented from various perspectives and with different criteria. Although the criteria of efficiency and effectiveness have gradually been challenged in favor of the criterion of democratic participation in policy design [28], designing an optimal network of financial protection for the poor in the health system in Iran can be seen and introduced as a manifestation of democratic and efficient network governance.

The aim of this study was to map an optimal policy network model for supporting the poor through gathering social data on the governance of the network to support the poor in the Iranian health system based on survey. This type of data gathering is established through social network analysis which has been previously used as a systematic method to describe and analyze the network governance among multiple stakeholders in health system. The model will be effective to overcome the challenges of overlapping organizational tasks and legal duties of different institutions.

## Method

### Data collection

The current research is a quantitative study that employs a social network analysis approach. Based on a network of legal responsibilities and research into the perspectives of experts in order to improve, the poor’s financial safety net was created. We attempted to prepare a questionnaire of institutional options to accurately draw the optimal network after designing questions related to the optimal arrangement and communication among mandated institutions.

The questionnaire was designed with the goal of resolving problems in the legal obligations network, clarifying ambiguous positions, avoiding conflicting tasks and rework, and improving relationships between institutions.

The questionnaire had three sections: demographic information, proposed institutional options, and a commenting section; if desired. A summary of the objectives along with the institutional options for drawing up the optimal network was sent to 30 experts on health financial protection, and they were requested to submit their comments within a maximum of 1 week. The results were reviewed and analyzed, and the model was developed based on those comments.

To assess the validity of the questions, the questionnaire has been presented to several experts in network analysis and social welfare policy. Then, the questionnaire was given to five experts as a pilot. The accuracy and validity of the questions were reviewed and corrected based on experts’ recommendations.

### Sampling

The research sample included 22 well-known experts who were purposefully selected from experts. They had high level of experience in financial support for the poor and had also relationships with different actors in organizational networks including Ministry of Health and Medical Education (MOHME), Ministry of Cooperatives, Labor and Social Welfare (MCLSW), Iranian Health Insurance Organization (IHIO), Iranian Red Crescent Society (IRCS), as insiders, and independent experts, as outsiders. We had equal number of insiders and outsiders in our expert body of research with 11 insiders from four network organizations and other experts from the outside.

Insiders provided detailed information about relationship among actors based on their direct experiences. Similarly, outsiders were beneficial due to their ability to view the entire network without direct involvement and, therefore, without conflict of interest biases. These two groups of informants, insiders and outsiders, provide us the opportunity of additional level of confirmation regarding network data, and also reducing possible biases. The combination of insiders and outsiders and relying on information from internal and external experts balances the biases, widens the perspectives, and validate methodology for incomplete SNA data [29].

### Data analysis

A two-dimensional matrix was used to record the data collected from the questionnaire; the selected institutions by the experts formed the matrix rows, and the policies and support programs formed the columns. The value of matrix cells indicates the number of tasks each organization performs for different programs. A value of 2 was assigned to the responsible institutions and a value of 1 was assigned to cooperator institutions. Then, a one-dimensional matrix of institutions was formed to determine which one has the most collaboration with other institutions and which one has more centrality, power and position due to more collaboration with others. UCINET software was used to enter data and data analysis and NetDraw software was used to draw networks and visual analysis.

Micro-indicators such as degree centrality (the identification of the network’s dominant actors) and betweenness centrality (the identification of the network’s dominant actors) (identification of actors that link between others) [30], Centrality of eigenvectors (the point having a lot of central neighbors) [31], were used to represent the location of institutions in the network and were normalized to lie between 0 and 1 [32]. as well as Bonacich power (calculated based on the centralization of connected points to somewhere between − 1 and + 1) [33, 34]. Several social network research metrics help to understand a system’s governance capacities. Density (the ratio of the number of available links to the total number of potential links in the network) is one of the characteristics of a system that can be converted into network governance metrics. The density measure’s value can range from 0 to 1, with 1 representing complete network density) [35], Geodesic distance (the number of relationships between two actors in the shortest possible stage, which is actually the most optimal or successful contact between two actors) and centralization (to the degree that cooperation in the network focuses on a limited number of actors rather than being equally distributed among all members) [36], In this network, both of them, which ranged from 0 to 1, were determined.

### Findings

The amount of network indicators of the institutions proposed by the experts, which has been calculated using UCINET software, is presented in Table 1. Only 11 of the 44 institutions listed in the network were recommended by the experts to be included in the optimal network. The optimal network has solved the problems in the network of legal obligations, such as the density of contact between certain central institutions and the isolation of other institutions, and relationships have been distributed in a fair and orderly manner among all institutions present in the network.

**Table 1:** Indicators of the optimal network of institutions related to financial protection for the poor

	Institution	Degree Centrality	Betweenness Centrality	Eigenvector Centrality	Bonacich’s powe*r*
1	Welfare Organization (WO)	0.372	0.825	0.486	1.611
2	Imam Khomeini Relief Committee	0.348	0.825	0.428	.1.419
3	Ministry of Cooperative, Labor and Social welfare (MCLSW	0.338	0.825	0.508	1.684
4	Iranian Health Insurance Organization (IHIO)	0.319	0.825	0.510	1.690
5	Charities	0.177	0.125	0.191	0.633
6	Ministry of Health and Medical Education (MOHME)	0.125	0.825	0.120	0.397
7	Municipalities	0.086	0.125	0.086	0.285
8	Supreme Council of Welfare and Social Security (SCWSS)	0.057	0	0.056	0.188
9	Iranian Red Crescent Society (IRCS)	0.045	0.125	0.043	0.144
10	Supreme Council of Health Insurance (SCHI)	0.024	0.500	0.018	0.059
11	Plan and Budget Organization (PBO)	0.009	0	0.008	0.028

**Fig. 1:**
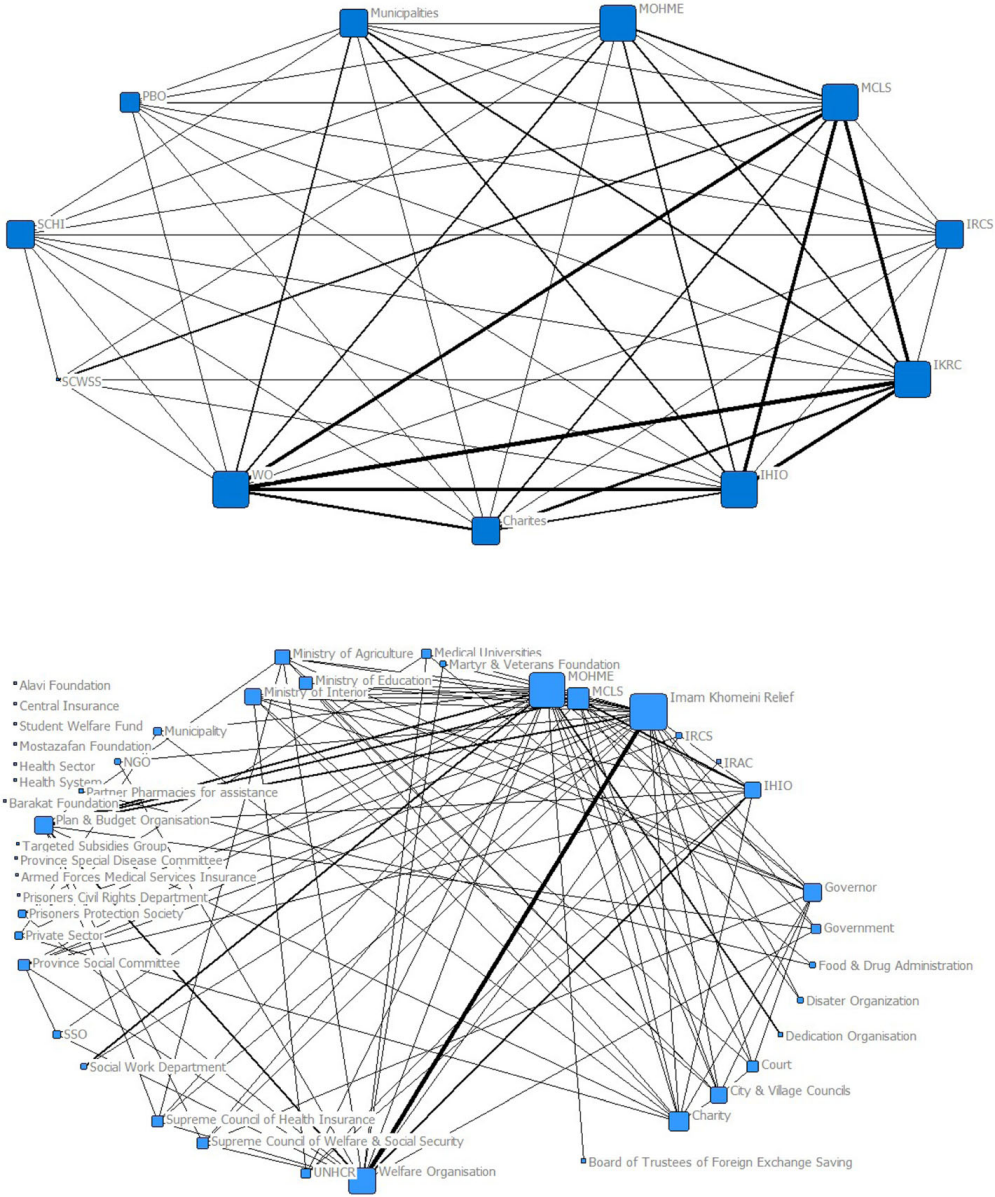
The degree centrality of the optimal financial protection network (above) and the legal obligations network of financial protection (below)

Legal collaboration between responsible and partner institutions regarding financial protection for the poor in two different optimal and legal networks is presented in Fig. 1. The intensity and weakness of communication and the number of common tasks between the two entities are indicated by the thickness of the lines, and organizations with a higher degree centrality are represented by large squares.

Strengthening and balancing relationship of coordinating bodies (including Supreme Council, Ministries and the Planning and Budgeting Organization) with executive bodies (Imam Khomeini Relief Foundation, Welfare Organization and Iranian Health Insurance Organization (IHIO) on the one hand, and adequate relationship among executive bodies with non-governmental institutions (NGO) on the other hand, shows that in the proposed model relationship is seen in the form of a network. The strong presence of NGOs reflects the institutional approach to policy-making for financial protection. Balanced distribution of power between these institutions reflects a network of relationships in financial protection policies.

### Network’s micro-indicators

As it shows in Table 1, according to the experts, Welfare Organization should have the most authority in the network and with a very small difference, Relief Foundation, MCLW and the IHIO stand in the next ranks. A noteworthy point is the high centrality of charities, which indicates the increased power of these institutions due to change their position and relations and a democratic approach to policy-making. The position of the Welfare Organization in the optimal network shows a greater tendency towards centrality than the Relief Foundation, which was the most powerful institution in the network of legal obligations.

**Fig. 2:**
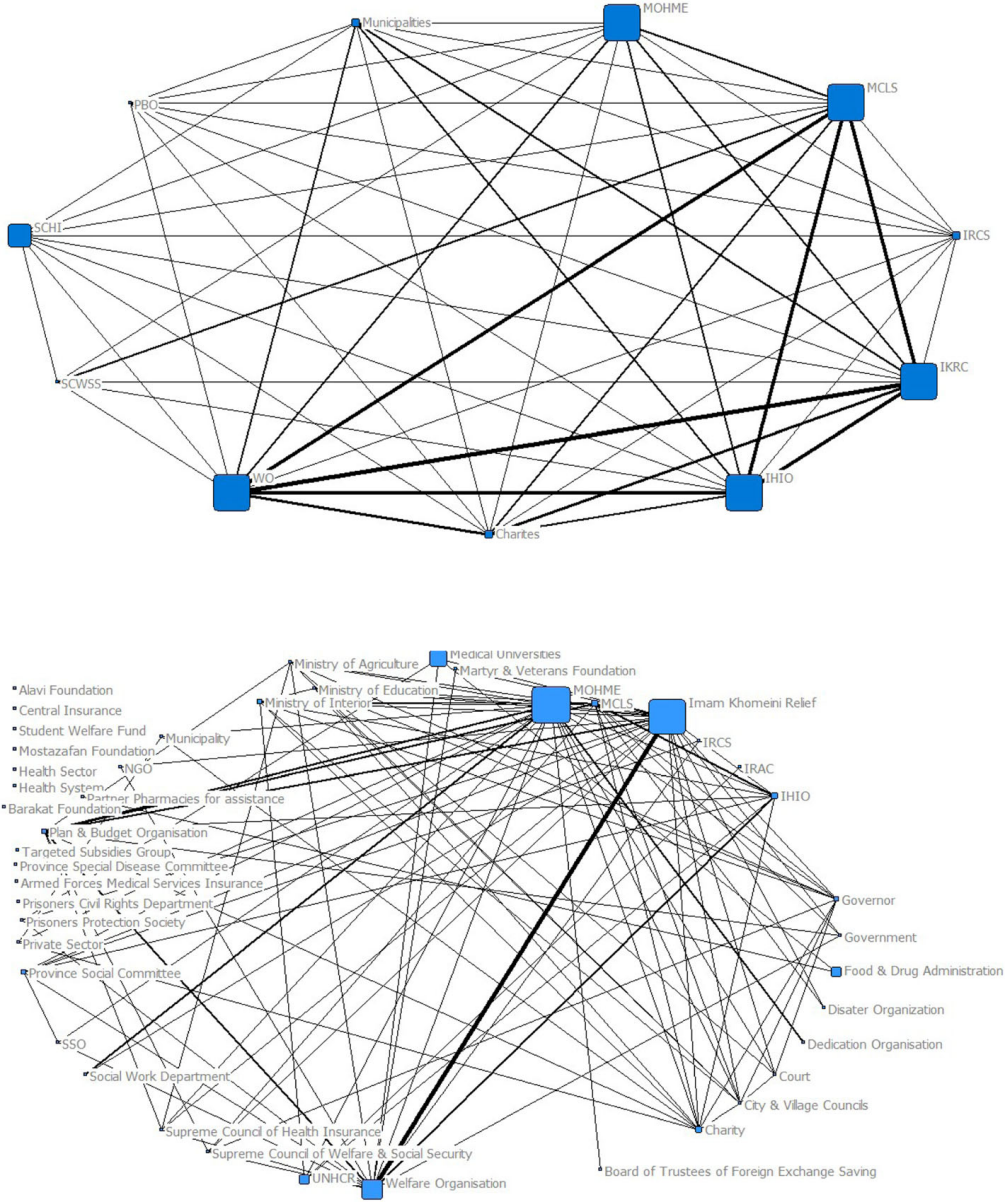
Betweenness centrality of the optimal financial protection network (above) and the legal obligations network of financial protection (below)

Betweenness centrality of the institutions is also of balanced distribution, and the Welfare Organization, the Relief Foundation, the MCLW, the MOHME, and the IHIO have been proposed with the most betweenness centrality. The high betweenness centrality of the Supreme Council of Health Insurance has made this council more accessible and more executive than the Supreme Council of Welfare and Social Security. Betweenness centrality distribution is shown in Fig. 2.

The IHIO and the MCLW have the most special eigenvector centrality due to the neighborhood with more central institutions, and the Welfare Organization is in the next rank. In the proposed network, the IHIO has the highest Bonacich power due to the centralities of the connected points, and the MCLW and the Welfare Organization are in the next ranks. It is understood that these institutions should have the most important role in formulating strategies as well as executive activities of financial protection.

**Fig. 3:**
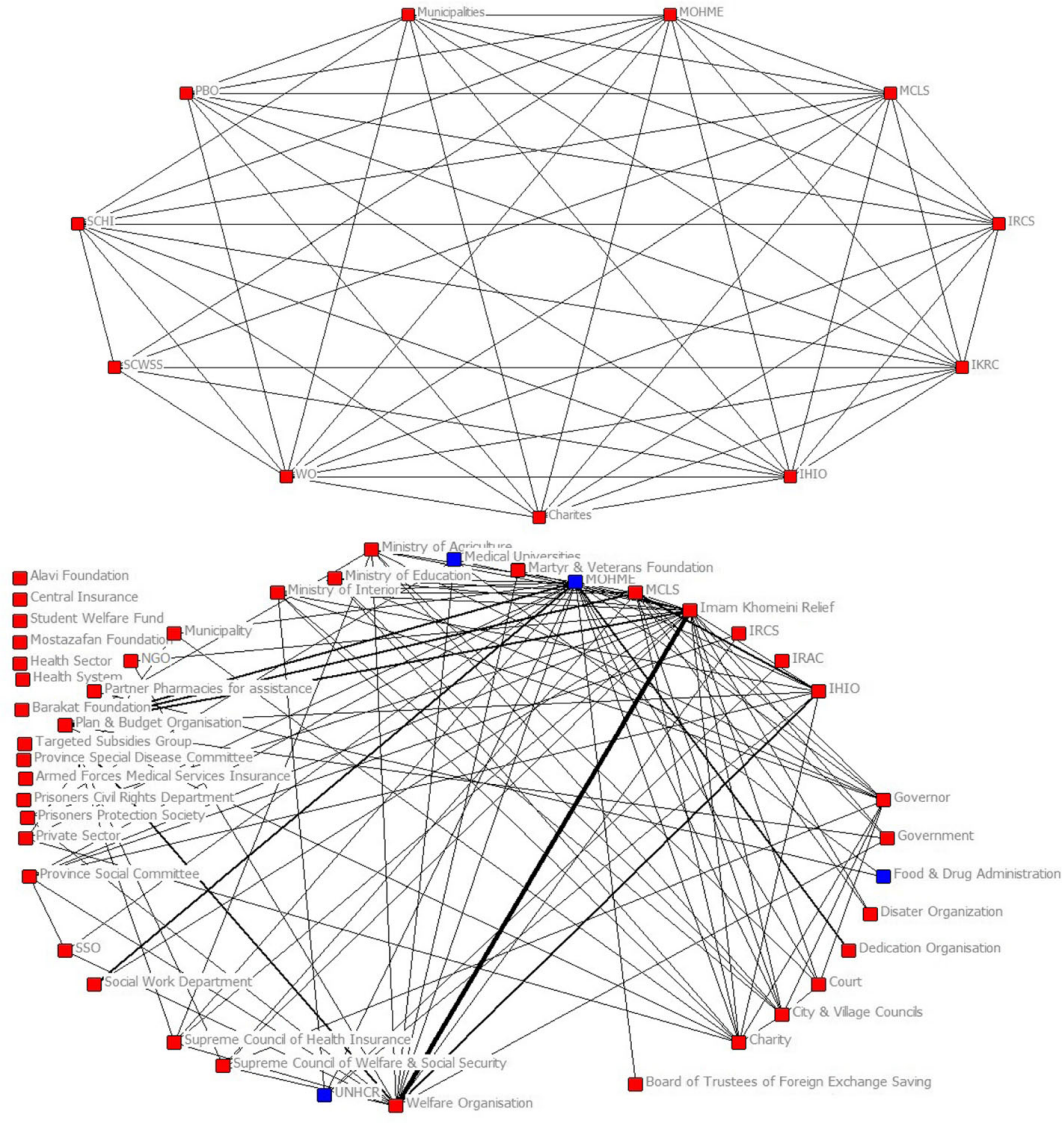
The cut points in the optimal network of financial protection (above) and the legal obligations network of financial protection (below)

In an optimal network, there are no cut points (entities whose removal from the network causes the network to become two separate parts and can cause or prevent relationship between other entities) and this indicates that the network is optimal in terms of more balanced distribution of power between institutions and better state of relationship between institutions. In general, due to the change in the position of institutions and increasing organizational relationships between them, the deep difference between the maximum and minimum number of relationships and the difference in the centrality of actors in the network of legal obligations has decreased. The cohesion has increased and the distribution of power and relationships has become more rational (Fig. 3).

Having more centrality means better position of accessibility in the network, which in turn increases collaboration power compared to others. In an optimal network of financial protection for the poor, the Welfare Organization, the Relief Foundation, the MCLW and the IHIO were recognized as the most powerful institutions. The majority of experts have chosen the following tasks and responsibilities for different institutions: the Supreme Council of Welfare and Social Security for policy-making and supervising financial protection policies; the MCLW and the Welfare Organization to identify the poor; the IHIO for basic insurance of the poor and cost-sharing coverage; the Welfare Organization and the Relief Foundation to cover the costs of services outside the benefit package and referring to health centers; and the MCLW as a single window for division of tasks among institutions. More network connections have been suggested between the Welfare Organization, the Relief Foundation, the MCLW and the IHIO.

### Network’s macro-indicators

Network governance indicators including density, centralization and distance between actors in the optimal network were calculated. The density of the legal obligations network of financial protection was 32.7%, which shows that the density among the 44 institutions present in the network is at a weak level and the relationship and collaboration between the institutions is not favorable. On the other hand, this index has reached 90% in the optimal network, which shows that the optimal network has a very high density.

The centralization in the optimal financial protection network is 0.11, which indicates that the network is decentralized and the flow of information is not restricted to a limited number of actors.

The average geodesic distance in the network of legal obligations is 2.2. It indicates that the organizational unity in the field of financial protection for the poor among the institutions involved in the network is weak. When all the actors are directly connected, the average distance will be 1 and the flow of information is expected to be fast. In the optimal network with fewer actors, the distance is 1.09, which indicates that the actors are all in direct contact with each other. The network is governed by a shared-governance model of the participants, according to these indicators.

## Discussion

Networks describe the pattern of relationship among actors, while the governance perspective on networks raises the question of how these networks achieve their goals and what mechanisms govern their performance. Where market mechanisms and hierarchies are not effective, the network as a structure of governance will be the most appropriate option [37].

In this article, to minimize the weaknesses of the legal obligations network, an optimal policy network has been proposed with regard to financial protection policies for the poor in order to access to the health services. We can design policies for financial protection of the poor in accessing health services more coherently through increasing cooperation and equitable distribution of power, as well as making partnerships more efficient, through a shared-governance model. The number of institutions responsible for financial protection of the poor in the proposed optimal network decreased from 44 to 11, and according to the experts, a large number of marginalized institutions were eliminated. The Welfare Organization and the Relief Foundation were designated by the experts as the most powerful institutions in the network.

There is pluralistic institutional diversity with different level of power in the existing network: Supreme Councils as regulation body, Ministry as headquarters, different organizations as the executive body, and para-governmental and non-governmental organizations, with the shared-governance arrangement, which ensures that all members have a fair say in decision-making.

Since power is distributed among different actors in the current network and the network is not focused on a single actor with unbalanced power, it cannot operate as a leading force. In proposed model all related organizations involved in financial protection for the poor have been seen in the network and therefore there is no need for creating a separate administrative organization.

Key predictors of the effectiveness of the optimal network governance indicate that there In the current network, there is no lead agency. This is due to the limited number of participants, high consensus on priorities, particularly due to the presence of several upstream laws on financial protection for the poor in the health system, and executive agencies, such as the IHIO, occupying the network’s central role, Relief Foundation and Welfare Organization, as well as organizations in higher administrative hierarchies in the network, such as ministries, have non-centralized positions, compared to executive organizations as an important indicator to determine the type of governance structure, which is clarified in this network [38].

As a result, shared-governance is the best network governance model for financial protection for the vulnerable in Iran’s health system. In this model the network is managed by engagement of all members in interaction, decisions are made collectively The power is equally distributed, and there are no cut points in the network, which can be achieved easily by two supreme councils as extra-ministerial institutions. This method of network governance is the most effective way to reach a solution to a complex problem.

Shared-governance is the most common and simplest form of network governance, especially in health sector. It is highly dense (high inter-organizational relationship) and decentralized, and rely on the collective participation of all organizations to make decisions and manage the network. Decision-making power is distributed among all organizations regardless of their size, resources and performance [39]. This type of governance creates a culture of participation among organizations and is therefore able to increase network integrity. This form of governance is important to promote the integration of service delivery organizations that serve heterogeneous target groups with a variety of problems [40].. Integrity is an essential feature of a network that determines the resource exchange among organizations and network’s response to the target groups. Matured shared-governance networks are more integrated than networks without shared-governance [41]. Due to the governance structure style, similarity of roles, and equal control, our proposed network appears to be highly integrated. Coordinative meetings -by sharing information, discussing new issues, exchanging resources, increasing collaboration, etc.- are effective tools for achieving shared-governance. The steering comity consisting of 4 or 5 representatives of the member organizations are in charge of the executive affairs. To increase network integrity, this type of governance must improve management strategies such as goal consensus, information exchange, and conflict resolution among organizations [42]. Meetings with members from the ideal network’s 11 member organizations may be convened with the aim of exchanging knowledge and formulating joint policy.

Since a simple form of governance is insufficient to solve a complex problem, and because of the economic crisis and funding issues that make it difficult to adequately help the vulnerable in accessing health services in Iran, the authors of this article propose that a network core group be created within the shared-governance framework. It will help more coherent decision-making. This core community includes Relief Foundation, Welfare Organization, MCLW, and IHIO, which are considered to have the most interactions with other members of the network, according to social network research. As Cristofoli study has shown, the success of shared-governance networks depends on the presence of network administrators and the use of formal inter-organizational coordination mechanisms. Successful shared-governance networks tend to take more bureaucratic approach to ensure power sharing and management of the network in accordance with established rules and regulations [43].

The study of the structure of Kerman Health and Food Security Council has shown that the shift of relationship from hierarchical and market-oriented to network logic has not yet occurred. This structure is suffered from problems such as large number of members, inability to solve complex problems, and lack of trust, legitimacy, and consensus among members. Therefore, a network management organization is considered as an effective way of governing to solve social problems in the field of health [44].

In legal obligation model, according to the law, several institutions are involved in policy making and policy implementation. This has led to overlapping tasks, slowness, and incoherence. The multiplicity of tasks and duties has created a tangle of confusion about the type, amount, and scope of responsibilities among the actors. Distribution of power among financial protection institutions for the poor is not well established, and there is no strong communication between governmental and non-governmental institutions, charities or private institutions in this field. These issues have been resolved in the optimal network with shared-governance model.

In the optimal network, charities’ position has been strengthened compared to the network of legal obligations. That is why experts in government consider charities as important bodies to support the poor in the health sector. Charities as non-profit sector are able to effectively address the problems of the poor with a more human-centered and flexible approach. They can reduce injustice and social deprivation [45]. Manenti states that NGOs and civil society are sometimes viewed negatively by government agencies and therefore their role becomes limited [46]. Our study find that Iranian experts have positive and participatory attitude to involve charities in supporting the poor.

Heo et al. used a network analysis approach to evaluate the relationship between government and non-government sectors and suggest policy-making options to promote the health of poor residents of disadvantaged areas. They showed that among different actors, community-based organizations (with spontaneously organized independent groups of volunteers for health promotion) have played a key role in sharing and controlling information resources for health promotion [47]; a role that can be played by charities in the optimal network of financial protection for the poor in Iran.

## Conclusion

Using a social network analysis approach as a policy tool to demonstrate the pattern of network governance of financial protection for the poor in the health system, this study showed that by strengthening the relationship between the main actors in this field, an organized system of network management with a limited number of coordinated actors can be achieved. The network has to include all actors from different levels; from policy-making to implementation in various fields, with clarifying tasks from identifying the poor to covering costs at the point of service delivery.

Social network analysis as a tool for displaying and manifesting network governance at the level of network management showed the relationship and proportionality between these two approaches in public policy sciences, including health policy. Governance in the optimal health financial protection network for the poor in Iran follows the shared-governance pattern due to its high density, low centralization and distance.

The network governance model governing the optimal policy-making network of financial support for poor people to access health services in Iran, as a model of transformation of inter-organizational communications in complex policy issues, can be the source of changes in the health governance system in Iran as a prerequisite for access to universal health coverage.

The current minimal literature primarily discusses governance issues without offering a straightforward solution. This study shed light on one of Iran’s most pressing issues in health system governance and attempted to find a solution by changing the existing governance system and introducing a new model to address the interorganizational challenges. In the case of financial protection for the poor in Iran, this paper added to our understanding of health system governance and its preferred framework, as well as to the literature by narrowly visualizing health system governance.

Recognizing the preferred governance model of organizations tasked with financial protection duties will encourage policymakers to use this proposed arrangement and bring these suggested actors to the negotiating table in order to design and enforce effective protection measures.

### Study strengths and limitation

To our knowledge, this is the first study in Iran to use quantitative methods, especially network analysis, to investigate the governance of institutions responsible for health financial protection. Other countries will benefit from the lessons and models discussed here. We had some limitations in addressing the shortcomings of literature in the form of peer-reviewed work derived from the Iranian context because the literature on the governance of health financial protection organizations in Iran was not rich and we found no research to compare this problem directly. This limitation could add to the significance and value of this study in filling an information gap. Because of political and institutional interests, some participants may have felt compelled to keep their technical opinions to themselves. We attempted to monitor this possible source of bias by using heterogeneous sampling and soliciting the views of various actors.

## Data Availability

The datasets used and/or analyzed during the current study are available from the corresponding author on reasonable request.
